# Multiple Omics Integration Reveals Key Circular RNAs in Hepatocellular Carcinoma

**DOI:** 10.3389/fonc.2021.621353

**Published:** 2021-05-18

**Authors:** Zi-Li Huang, Xiu-Yan Huang, Jin Huang, Xin-Yu Huang, Yong-Hua Xu, Jian Zhou, Zhao-You Tang

**Affiliations:** ^1^ Department of General Surgery, Shanghai Jiaotong University Affiliated Sixth People’s Hospital, Shanghai, China; ^2^ Department of Radiology, Xuhui District Central Hospital of Zhongshan Hospital, Fudan University, Shanghai, China; ^3^ Department of Pathology, Shanghai Jiaotong University Affiliated Sixth People’s Hospital, Shanghai, China; ^4^ Liver Cancer Institute and Zhongshan Hospital, Fudan University, Shanghai, China

**Keywords:** hepatocellular carcinoma, circular RNAs, mTOR signaling pathway, gluconeogenesis, HNF3A pathway

## Abstract

**Background:**

HCC is one of the most common malignancies with an increasing incidence worldwide, especially in Asian countries. However, even though targeted cancer therapy drugs such as sorafenib and regorafenib are available, the overall outcome of HCC remains unsatisfactory. Thus, it is urgent to investigate the molecular mechanisms of HCC progression, so as to provide accurate diagnostic criteria and therapeutic targets.

**Methods:**

RNA-seq data was used to identify and quantify circular RNAs (circRNAs). DESeq2 was used to identify the differentially expressed circRNAs. miRNA binding sites within circRNAs were identified by miRanda. Gene set enrichment analysis (GSEA) was conducted to predict the biological function of circRNAs.

**Results:**

The differential expression analysis identified 107 upregulated and 95 downregulated circRNAs in HCC tissues. We observed that a differentially expressed circRNA (DE-circRNA), hsa_circ_0141900 was highly negatively correlated with its parental gene *RAB1A* (PCC < -0.6), which was also closely associated with mTOR signaling pathway. Moreover, we also constructed competing endogenous RNA (ceRNA) network to identify key circRNAs involved in HCC. Notably, hsa_circ_0002130 and hsa_circ_0008774 were highly correlated with the genes involved in gluconeogenesis and HNF3A pathway *via* the target genes, *GOT2* and *AR*, suggesting that the two circRNAs might regulate these pathways, respectively. Survival analysis revealed that *GOT2* was associated with favorable prognosis. Furthermore, high expression of hsa_circ_0002130 was found to inhibit tumor cell growth and promotes GOT2 expression.

**Conclusion:**

In summary, the circRNAs highlighted by the integrative analysis greatly improved our understanding of the underlying mechanism of circRNAs in HCC.

## Introduction

Hepatocellular carcinoma (HCC) is one of the most common malignancies with an increasing incidence worldwide (1, 2). Recognized risk factors for HCC include cirrhosis, HBV and/or HCV infection, and obesity (3). An individual with cirrhosis is considered to be at higher risk of HCC, and increased levels of alpha fetoprotein (AFP) often serves as clinical biomarker for HCC diagnosis, yet its sensitivity is not very promising (4). In fact, almost two-third of HCC patients are diagnosed at advanced stages, where most therapies seldom yield satisfying outcomes (5, 6). Thus, it is urgent to investigate the molecular mechanisms of HCC progression, so as to provide accurate diagnostic criteria and therapeutic targets.

With the advances in microarray and RNA sequencing technologies, novel associations between non-coding RNAs (ncRNAs) and the development of tumor have been unveiled. Previous study has identified 525 and 323 lncRNAs as recurrently downregulated or upregulated in HCC patients (1). According to previous studies, lncRNA-activated by TGF-β (lncRNA-ATB) has the ability to upregulate ZEB1 and ZEB2, and predispose HCC patients to metastases, while lncRNA downregulated in liver cancer stem cells (lnc-DILC) functions to suppress the expression of inflammatory cytokine IL−6, which in turns inhibits IL6-STAT3 autocrine signaling and suppress LCSC propagation (7, 8). LncRNA is also closely related to splicing dysregulation in HCC. For example, by inducing exon 4 inclusion in lncRNA-PXN-AS1, one oncofetal splicing factor MBNL3 upregulates PXN to promote HCC tumorigenesis (8). Other than lncRNA, circRNA, a novel class of endogenous noncoding RNA, also plays a critical role in the development of HCC, as it owns microRNA sponging properties and has exhibited aberrant expression in various tumors (9). circHIPK3 could sponge to 9 miRNAs, including miR124, miR-152, miR-193a, miR-29a, miR-29b, miR-338, miR-379, miR-584 and miR-654, and circRNA-ITCH was found to sponge to miR-7, miR-17, and miR-214 (9). While these circRNAs and their targets are greatly associated with tumorigenesis and progression, the underlying mechanism is yet not fully appreciated. In the present study, we aimed to discover the key circRNAs involved in HCC, and annotated their biological functions or signaling pathways. The circRNAs highlighted by the integrative analysis greatly improved our understanding of the underlying mechanism of circRNAs in HCC.

## Materials and Methods

### Gene and miRNA Expression Quantification

The RNA sequencing files of non-tumor, HCC, and portal vein tumor thrombosis (PVTT) were downloaded from Sequence Read Archive (SRA) database (10) with accession SRP069212 (1), the RNA library of which was constructed by rRNA depletion and could be used for circRNA detection. The SRA files were first uncompressed by fastq-dump. The resulting fastq files were then mapped to human reference genome with GENCODE (11) gene annotation v19 by hisat2 (12). The gene expression levels were quantified by StringTie (13). The small-RNA-seq data were preprocessed by Trimmomatic (14), and the miRNA prediction and quantification were implemented by miRDeep2 (15).

### Circular RNA Detection and Quantification

To detect the circular RNAs (circRNAs), we mapped to reads to human reference genome (UCSC hg19) using BWA (16). The circular RNAs were detected by the CIRI tool (17), which used the GENCODE gene annotation v19. The circRNA expression levels were estimated by the number of reads that support the splicing junction sites.

### Differential Expression Analysis

The differential expression analysis was implemented in R/Bioconductor DESeq2 package (18), which normalized the read counts of the genes or circRNAs and tested the difference by Ward test. The ratio method was used to estimate the size factor. The parametric type was used to fit a dispersion-mean. The genes or circRNAs were deemed as differentially expressed if adjusted P-value < 0.05 and fold change >2 or < 1/2 were used as the thresholds.

### Gene Set Enrichment Analysis (GSEA)

To conduct GSEA for circRNA annotation, the Pearson correlation coefficients (PCC) between a given circRNA and all genes were first calculated. The pre-ranked genes based on the PCC were then used to identify pathways enriched by the highly correlated genes. The gene sets with gene numbers between 5 and 500 were used. A total of 1000 times permutation was used for the P-value calculation. The GSEA was implemented in R/Bioconductor fgsea package (19).

### Prediction of miRNA Binding Sites Within Junction Regions of circRNAs

The miRNA binding sites within the junction regions (23-bp flanking the splicing junction sites) of circRNAs were predicted by miRanda software (20) with score cutoff ≥ 140, energy cutoff ≤ −20 kcal/mol. In addition, the correlation analysis of the miRNA and circRNA expression was used to determine their inverse relationship (PCC < -0.35).

### Cell Culture, Vector Transfection, and RNA Extraction

The human HCC cell lines, MHCC97H and Hep3B, were cultured in RPMI-1640 medium supplemented with 10% fetal bovine serum (Gibco) and 1% penicillin–streptomycin. The cells were incubated at 37°C with 5% CO_2_. Hsa_circ_0002130 overexpression vector was synthesized *via* cloning hsa_circ_0002130 sequence into pcDNA3.1 circRNA mini vector, with the pcDNA3.1 circRNA mini vector (Addgene, Cambridge, MA, U.S.A.) as negative control (pcDNA). The cell transfection was conducted using Lipofectamine 2000 (Thermo Fisher) for 24 h. Total RNAs were extracted from the cells using a TRizol reagent (Invitrogen, Carlsbad, CA, USA).

### The Quantitative Reverse-Transcription Polymerase Chain Reaction (qRT-PCR)

Reverse transcription was performed by the PrimeScript RT reagent kit (TaKaRa, Tokyo, Japan) following the manufacturer’s protocol. RNA expression was quantitatively analyzed using an ABI Prism 7900HT (Applied Biosystems, Foster City, CA). GAPDH as an internal reference for GOT2 and hsa_circ_0002130. The primers for GOT2: forward: 5’- AAGAGTGGCCGGTTTGTCAC-3’, reverse: 5’-AGAAAGACATCTCGGCTGAACT-3’; The primers for hsa_circ_0002130: forward: GTAACCTGGATGAGGACATC; reverse: CTTTCTTGTCCGACATGCTC; The primers for GAPDH: forward: 5’-GAAAGCCTGCCGGTGACTAA-3’, reverse: 5’-TTCCCGTTCTCAGCCTTGAC-3’. All these experiments were conducted in triplicates.

### Cell Counting Kit-8 (CCK8) Assay

The cell proliferation levels were detected by Cell Counting Kit-8 (CCK8) assay. The experiments were conducted in 96-well plates with 2 × 10^3^ cells/well. Using a microplate reader (Bio-Rad, Shanghai, China), we detected the absorbance at 450 nm following the manual. All these experiments were conducted in triplicates.

### Western Blotting

Western blotting was conducted to detect the protein levels of GOT2 (1:1000, Proteintech) and GAPDH (1:1000, Proteintech) according to our previous reports (21, 22).

### Survival Analysis

The survival analysis was implemented in R survival package. The samples were stratified into two groups, high and low expression groups, based on the median of expression values for each gene. Log-rank test was used to test the survival difference between the two groups.

## Results

### Overview of the Circular RNAs in Non-Tumor Tissues, Tumors and PVTT

**Figure 1:**
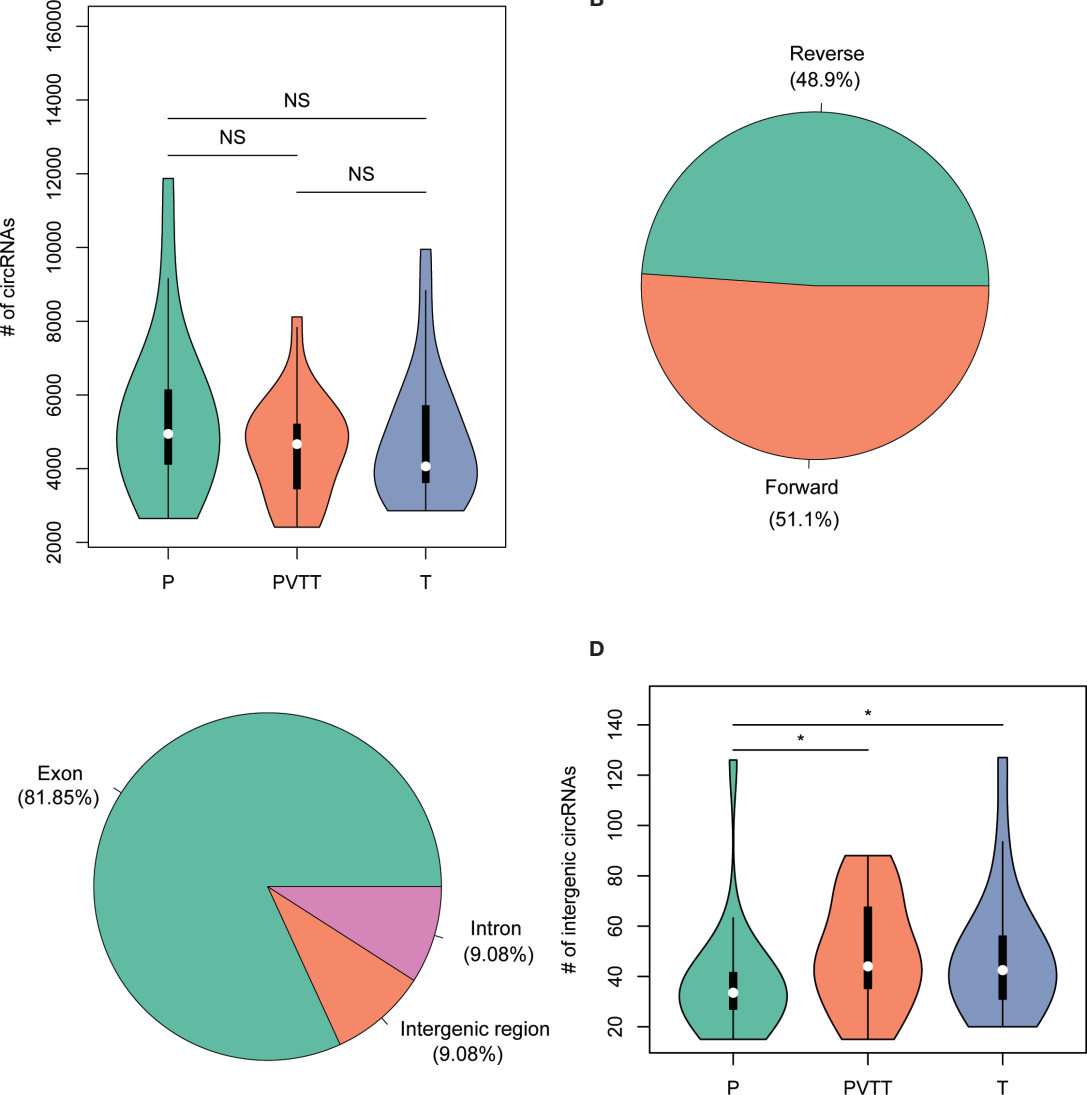
The characteristics of the circRNAs identified by RNA-seq data. **(A)** The distribution of the number of circRNAs in non-tumor (P), PVTT (PVTT), and HCC (T). **(B)** The proportion of strands that the circRNAs were originated from. **(C)** The proportion of the genomic regions that the circRNAs were originated from. **(D)** The distribution of the number of intergenic circRNAs with high confidence in non-tumor (P), PVTT (PVTT), and HCC (T). *P-value < 0.05, ns, not significant.

To detect the circular RNAs (circRNAs) in HCC and non-tumor tissues, we collected RNA sequencing data from matched non-tumors, tumors, and portal vein tumor thrombosis (PVTT, a main route for intra-hepatic or distant metastases of HCC) of 20 HCC patients to identify the reads that support the junction sites of the circRNAs. Totally, we identified 43,744 circRNAs in all samples. Specifically, there were 25,182, 27,135, and 26,232 circRNAs detected in non-tumors, tumors and PVTT, respectively. However, the difference of the number of circRNAs were not significant between the three types of tissues (*P* > 0.05), with medians of 4944.5, 4062.5, and 4666 in non-tumors, tumors and PVTT (**Figure 1A**). Overall, the percentage of circRNAs originated from the forward strand was about 51.1%, which was very close to a 50% probability (**Figure 1B**). Moreover, the proportion of the circRNAs located within the exonic regions was about 81.85%, followed by intergenic and intronic regions (**Figure 1C**). The intergenic circRNAs with high confidence (# junction reads >5) was observed more in both tumors and PVTT than non-tumors (*P* < 0.05, **Figure 1D**). These results unveiled that circRNAs were abundant in liver tissues, and those originated from noncoding regions might play critical roles in the tumorigenesis or progression of HCC.

### Differentially Expressed circRNAs Between HCC and Non-Tumor Tissues

**Figure 2:**
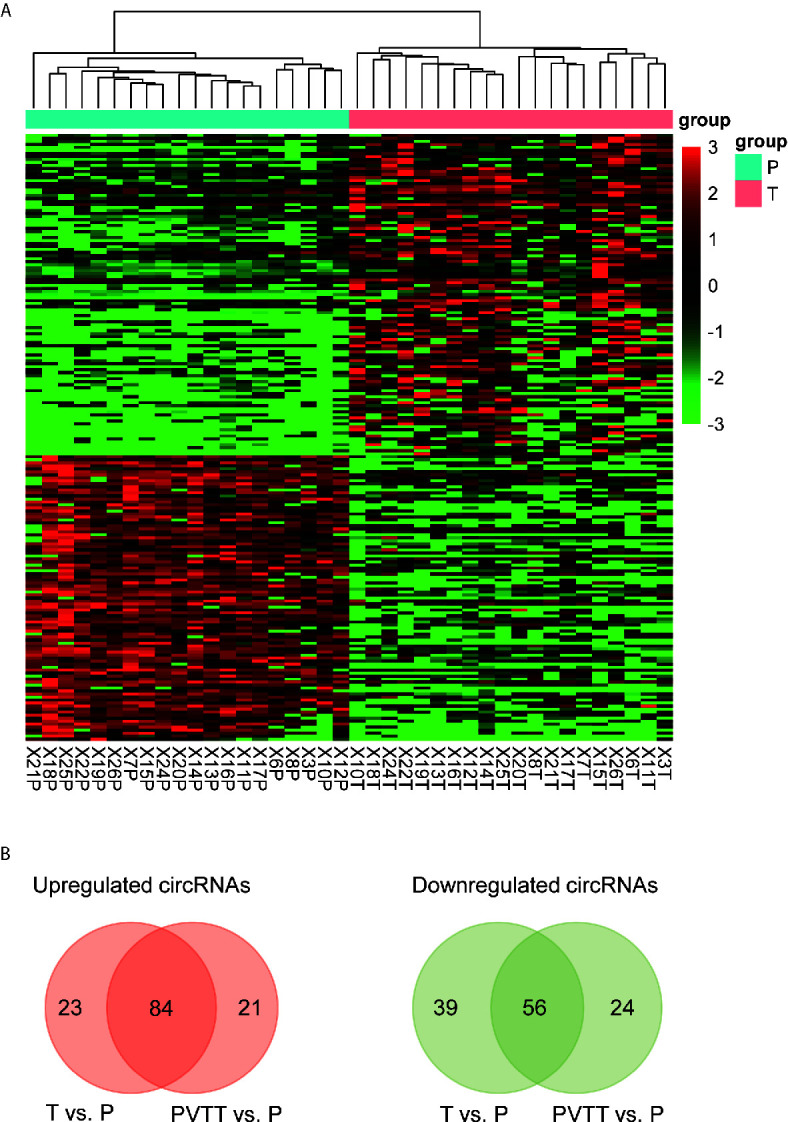
Differentially expressed circRNAs in HCC. **(A)** The expression patterns of the differentially expressed circRNAs. **(B)** The upregulated and downregulated circRNAs that also detected to be dysregulated in PVTT.

To identify the differentially expressed (DE) circRNAs, we conducted differential expression analysis of the circRNAs with high confidence (# junction reads >5) using R/Bioconducter DESeq2 package. Specifically, we identified 107 upregulated and 95 downregulated circRNAs in HCC tissues. The hierarchical clustering analysis of these DE-circRNAs suggested that these dysregulated circRNAs were capable of differentiating the HCC and non-tumor tissues (**Figure 2A**). Moreover, most of the upregulated and downregulated circRNAs were also observed to be differentially expressed in PVTT as compared with the non-tumor tissues (**Figure 2B**, hypergeometric test, P<0.05), suggesting that most of these dysregulated circRNAs in HCC may also be associated with HCC metastasis. The top-five upregulated and downregulated circRNAs were shown in **Table 1**. Notably, the circRNA, hsa_circ_0001727, originated from *ZKSCAN1* was reported to inhibit hepatocellular carcinoma cell growth, migration, and invasion through several cancer-related signaling pathways (23). In addition, circRNA-100338 (hsa_circ_0000130), which was identified to be upregulated in HCC by our previous studies (21, 22, 24), was observed to be upregulated in this HCC cohort (**Table 1**, *P* < 0.05). These results suggested that the differential expression analysis could effectively identify the dysregulated circRNAs.

**Table 1:** The top-five upregulated and downregulated circRNAs in HCC.

circRNA ID	circRNA type	Symbol	Strand	log2 Fold Change	Stat.	P value	Padj.
hsa_circ_0016599	exon	*DNAH14*	+	6.06	9.39	5.74E-21	1.21E-17
hsa_circ_0084615	exon	*ASPH*	–	2.60	8.21	2.30E-16	4.83E-13
hsa_circ_0016600	exon	*DNAH14*	+	2.54	7.57	3.85E-14	8.10E-11
hsa_circ_0016601	exon	*DNAH14*	+	2.62	7.55	4.41E-14	9.28E-11
hsa_circ_0000130	exon	*SNX27*	+	1.22	7.50	6.26E-14	1.32E-10
hsa_circ_0001306	intron	*RAD54L2*	+	-2.86	-8.54	1.35E-17	2.83E-14
hsa_circ_0001727	exon	*ZKSCAN1*	+	-1.70	-8.18	2.74E-16	5.76E-13
hsa_circ_0007619	exon	*LARP1B*	+	-2.15	-7.32	2.53E-13	5.30E-10
hsa_circ_0008844	exon	*MFSD2A*	+	-5.17	-7.12	1.11E-12	2.33E-09
chr12:96381971|96384310 (circHAL)	exon	*HAL*	–	-3.85	-6.93	4.12E-12	8.63E-09

Stat, Statistic; Padj, Adjusted P-value.

### The Association Between the DE-circRNAs and Their Parental Genes

**Figure 3:**
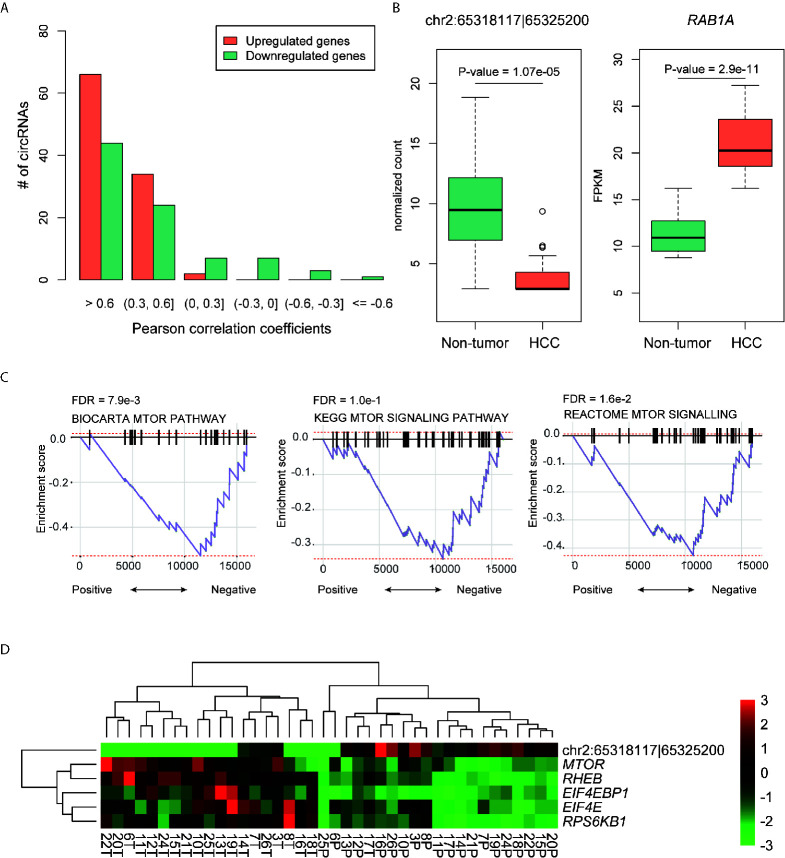
The DE-circRNAs and their parental genes. **(A)** The distribution of the Pearson correlation coefficients between the DE-circRNAs and their parental genes. **(B)** The expression patterns of hsa_circ_0141900 and RAB1A in HCC and non-tumors. **(C)** The significance of the mTOR signaling pathway enriched by the highly correlated genes with hsa_circ_0141900 by GSEA. **(D)** The expression profiles of the highly correlated genes with hsa_circ_0141900in mTOR signaling pathway.

To explore whether the DE-circRNAs were associated with their parental genes, we conducted correlation analysis on each pair of DE-circRNA and corresponding parental gene. We found that most of the DE-circRNAs were positively correlated with their parental genes (**Figure 3A**). Notably, 66 upregulated and 44 downregulated circRNAs had high correlation (Pearson correlation coefficient, PCC > 0.6), which accounted for 64% and 51% of these circRNAs, respectively.

Moreover, we also observed a DE-circRNA, hsa_circ_0141900, with high negative correlation (PCC < -0.6). The DE-circRNA, hsa_circ_0141900 and its parental gene, *RAB1A*, were downregulated and upregulated in HCC (**Figure 3B**), respectively. The negative correlation between hsa_circ_0141900 and parental gene *RAB1A* indicated that the circRNA hsa_circ_0141900 may have the potential to suppress RAB1A linear RNAs in isoform switch manner. As RAB1A was involved in mTOR signaling pathway (25), we also investigated whether the dysregulation of hsa_circ_0141900 and *RAB1A* were associated with mTOR signaling pathway. We found that the expression of hsa_circ_0141900 was highly correlated with mTOR signaling pathway by gene set enrichment analysis (GSEA, FDR < 0.1, **Figure 3C**). Specifically, we also observed the key regulators in mTOR signaling pathway, such as *MTOR, RHEB, EIF4EBP1, EIF4E, RPS6KB1*, were highly correlated with hsa_circ_0141900 (PCC < -0.6), further suggesting that hsa_circ_0141900 was highly associated with mTOR signaling pathway.

### Identification of DE-circRNAs Acting as Competing Endogenous RNAs

To identify the DE-circRNAs acting as competing endogenous RNAs (ceRNAs), we designed a data analysis pipeline by integrating the miRNA-mRNA, miRNA-circRNA interactions, with the expression patterns of mRNAs, miRNAs and circRNAs. We first predicted the miRNA binding sites of the circRNAs within the junction regions by miRanda, and collected mRNA-miRNA interactions from miRTarBase database (26).

**Figure 4:**
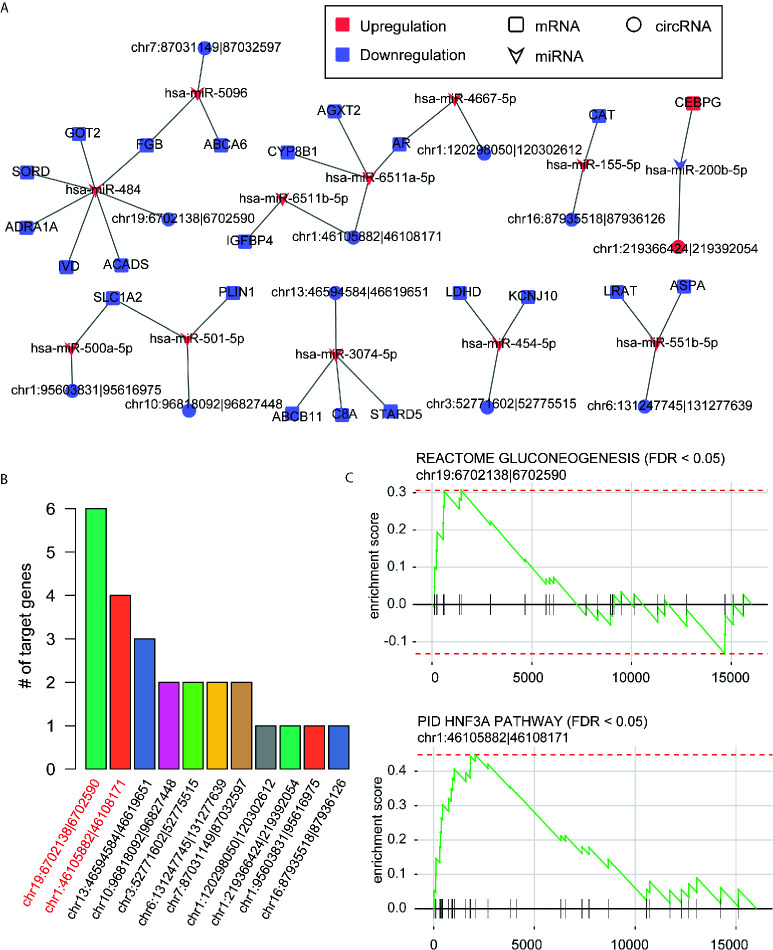
The competing endogenous RNA (ceRNA) network and functional annotation of the key circRNAs. **(A)** The ceRNA network constructed by the miRNAs, differentially expressed genes and circRNAs. **(B)** The number of target genes of circRNAs in the ceRNA network. **(C)** The predicted pathways that the hsa_circ_0002130 and hsa_circ_0008774 may participate in.

Given a stringent threshold -0.35 for PCC of miRNA-mRNA and miRNA-circRNA, we predicted 25 miRNA-mRNA and 12 miRNA-circRNA interactions, which constructed the ceRNA network. As shown in **Figure 4A**, the ceRNA network consisted of 12 miRNAs, 22 mRNAs, and 11 circRNAs. Remarkably, most of the circRNAs involved in the ceRNA network were downregulated in HCC, while only one circRNA, hsa_circ_0004314, in the network was upregulated. Among the circRNAs in the ceRNA network, hsa_circ_0002130 had the greatest number of target genes/mRNAs, followed by hsa_circ_0008774 (**Figure 4B**). To predict the biological pathways that the circRNAs may participate in, we also conducted gene set enrichment analysis (GSEA) of the pathways that the target genes of the circRNAs were involved in. We observed that hsa_circ_0002130 and hsa_circ_0008774 were highly correlated with the genes involved in gluconeogenesis and HNF3A pathway (**Figure 4C**), suggesting that the two genes might regulate these pathways *via* the target genes, *GOT2* and *AR*, respectively. These results highlighted two circRNAs and their pathways in the ceRNA network.

### The Prognostic Values of the Target Genes of circRNAs

**Figure 5:**
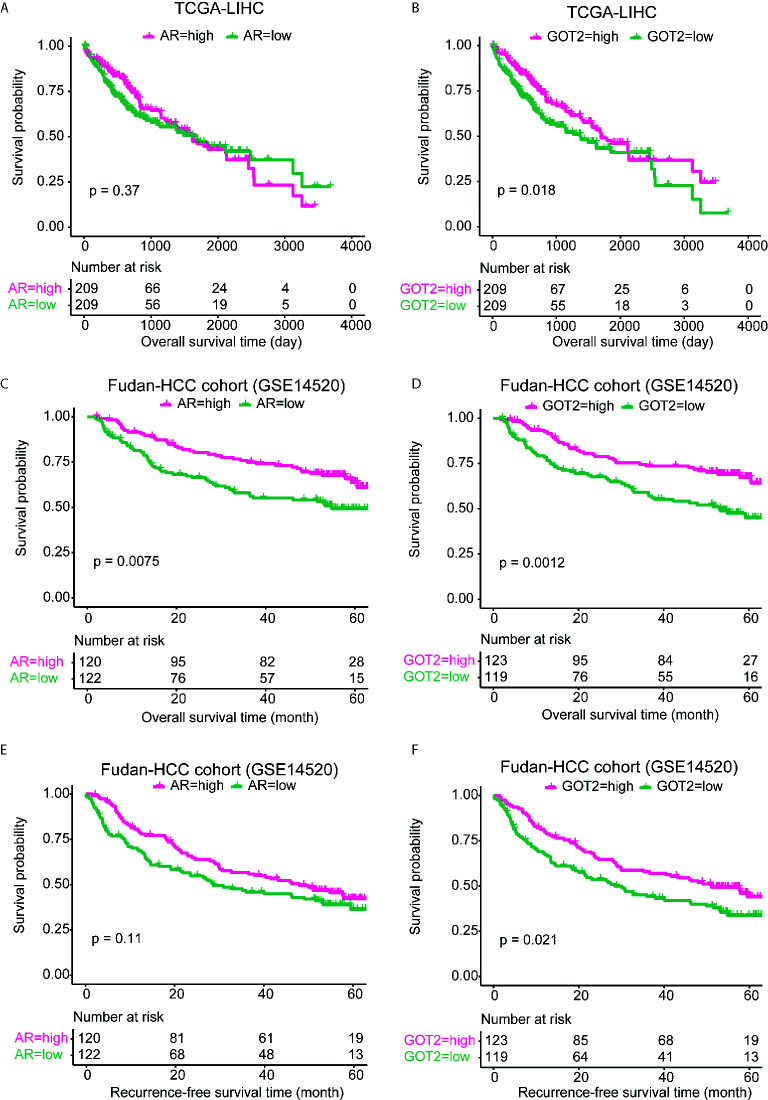
Kaplan–Meier curves for AR and GOT2 in TCGA-LIHC and Fudan-HCC cohorts. The overall survival analyses of AR and GOT2 in TCGA-LIHC cohort were displayed in **(A, B)**. The overall and progression-free survival analyses of the two genes in Fudan-HCC cohort were displayed in **(C, D)** and **(E, F)**, respectively.

We evaluated the prognostic significance of circRNA target genes in the ceRNA network due to the lack of HCC circRNA expression data with long-term follow-up and large sample size, which could indirectly indicate the prognostic values of the circRNAs. The samples were first stratified into two groups based on the expression value of *GOT2* and *AR*, respectively. We observed that *GOT2*, rather than *AR*, was associated with long overall survival in TCGA-LIHC cohort (**Figures 5A, B**), which was consistent with its downregulation in HCC. However, in the Fudan-HCC cohort, the two genes were both associated with longer progression-free survival (**Figures 5C, D**). In accordance with the TCGA-LIHC cohort, *GOT2*, but not *AR*, was significantly associated with overall survival in Fudan-HCC cohort (**Figures 5E, F**, P<0.05). Furthermore, we also built multivariable Cox models using TNM stage, BCLC stage, cirrhosis, and AFP as co-factors. Consistently, low expression of GOT2 was still highly correlated with overall survival or recurrence-free survival (**Supplementary Table 1**), suggesting that GOT2 expression is an independent prognostic factor in HCC. The close association between the circRNA target genes and overall or progression-free survival suggested that the two circRNAs, hsa_circ_0002130 and hsa_circ_0008774, might also be associated with prognosis of HCC patients.

### The High Expression of hsa_circ_0002130 Inhibits Tumor Cell Growth and Promotes GOT2 Expression

**Figure 6:**
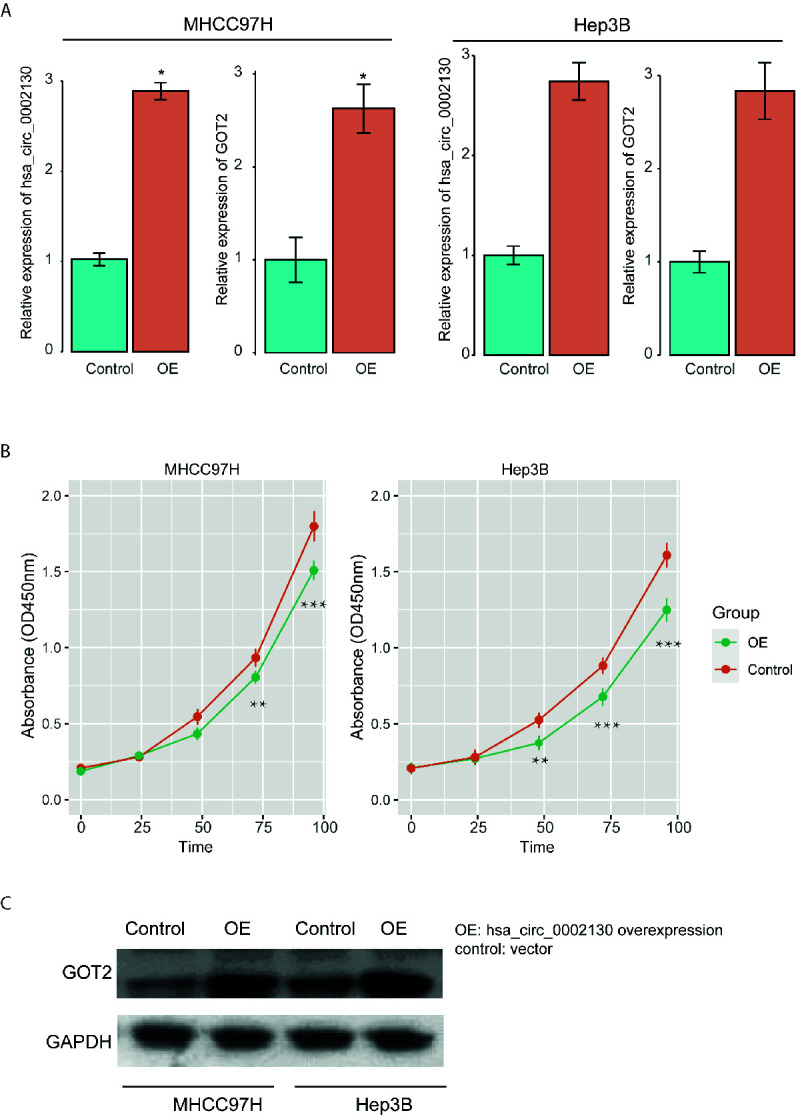
The impact of hsa_circ_0002130 on tumor growth and GOT2 expression. **(A)** The RNA expression levels of hsa_circ_0002130 and GOT2 in the cell lines with (OE) and without (control) hsa_circ_0002130 overexpression. **(B)** The CCK-8 assay of the cell lines with (OE) and without (control) hsa_circ_0002130 overexpression. **(C)** The protein expression of GOT2 in OE and control groups. "*", "**", and "***" indicate the P-value lower than 0.05, 0.01, and 0.001, respectively.

To further investigate the impact of hsa_circ_0002130 on tumor cell growth and expression of its target gene, we overexpressed its expression in two human HCC cell lines (MHCC97H and Hep3B). Specifically, hsa_circ_0002130 was significantly upregulated in cells with hsa_circ_0002130 overexpression as compared with the controls using qPCR method (**Figure 6A** and **Supplementary Table 2**). The CCK-8 assay revealed that the proliferation levels of HCC cell lines were significantly suppressed by hsa_circ_0002130 overexpression (**Figure 6B** and **Supplementary Table 2**). Moreover, we also observed that both RNA and protein expression of GOT2 were upregulated in cell lines with hsa_circ_0002130 overexpression (**Figures 6A, C**). These results indicated that high expression of hsa_circ_0002130 could significantly inhibit tumor cell growth and promote GOT2 expression in HCC.

## Discussion

HCC is one of the most common malignancies with an increasing incidence worldwide, especially in Asian countries. However, even though targeted cancer therapy drugs such as sorafenib and regorafenib are available, the overall outcome of HCC remains unsatisfactory. Thus, it is urgent to investigate the molecular mechanisms of HCC progression, so as to provide accurate diagnostic criteria and therapeutic targets. In this study, we detected and quantified the circRNAs in HCC and non-tumor tissues using RNA sequencing data collected from SRA database. Among the circRNA identified by RNA-seq data, the intergenic circRNAs with high confidence (# junction reads > 5) was observed more in both tumors and PVTT than non-tumors (*P* < 0.05, **Figure 1D**), suggesting that circRNAs originated from noncoding regions might play key roles in HCC. The differential expression analysis identified 107 upregulated and 95 downregulated circRNAs in HCC tissues. Notably, the circRNA, hsa_circ_0001727, originated from *ZKSCAN1* was reported to inhibit hepatocellular carcinoma cell growth, migration, and invasion through several cancer-related signaling pathways (23), suggesting that the differential expression analysis could effectively identify the dysregulated circRNAs.

To further explore the functional roles of DE-circRNAs in HCC, we investigated the association between the DE-circRNAs and their parental genes and ceRNA network. Most of the DE-circRNAs were positively correlated with their parental genes (**Figure 3A**). Notably, 66 upregulated and 44 downregulated circRNAs had high correlation (Pearson correlation coefficient, PCC > 0.6). However, a DE-circRNA, hsa_circ_0141900 was observed to be highly negatively correlated with its parental gene *RAB1A* (PCC < -0.6). As *RAB1A* was involved in mTOR signaling pathway (25), we also investigated whether the dysregulation of hsa_circ_0141900 and *RAB1A* were associated with mTOR signaling pathway (FDR < 0.1, **Figure 3C**), further suggesting that hsa_circ_0141900 was highly associated with mTOR signaling pathway.

Furthermore, as the circRNAs generally acted as ceRNAs by competing for the miRNAs with mRNAs (27, 28), we constructed ceRNA network by integrating miRNAs binding sites, and expression profiles of miRNAs, mRNAs and circRNAs. We finally predicted 25 miRNA-mRNA and 12 miRNA-circRNA interactions, which constructed the ceRNA network. Among the circRNAs in the ceRNA network, hsa_circ_0002130 and hsa_circ_0008774 were highly correlated with the genes involved in gluconeogenesis and HNF3A pathway (**Figure 4C**), suggesting that the two genes might regulate these pathways *via* the target genes, *GOT2* and *AR*, respectively. As metabolic reprogramming was considered as one of the hallmarks of HCC, more importantly, gluconeogenesis, an essential metabolic process for hepatocytes, was downregulated in hepatocellular carcinoma (29), suggesting that hsa_circ_0002130 might regulate gluconeogenesis *via GOT2*. Therefore, we speculated that the circRNA hsa_circ_0002130 might act as a miRNA sponge to increase the expression of GOT2, thereby inhibiting the tumor cell growth *via* gluconeogenesis. Moreover, Androgen receptor (AR) signaling played important roles in normal liver function and in progression of liver diseases (30), the downregulation of *AR* in the hsa_circ_0008774-related ceRNA network indicated that hsa_circ_0008774 may be the upstream regulator of AR signaling. In addition, we also investigated the prognostic values of these circRNA target genes, *AR* and *GOT2*, which indirectly indicated the prognostic significance of the circRNAs. Remarkably, *GOT2* was associated with favorable prognosis. To our knowledge, the prognostic value of *GOT2* has not been reported by previous studies. However, the function roles of the two genes have been widely reported in HCC (30–33). Furthermore, we also found that hsa_circ_0002130 could efficiently inhibit tumor cell growth and promote GOT2 expression, further suggesting that hsa_circ_0002130 might act as a tumor suppressor in HCC.

In addition, this study still had some limitations. First, the circRNAs identified by the RNA-seq data should be validated by more evidences. Second, the prognostic value of the circRNAs should be explored using the circRNA expression data. Third, small proteins encoded by some circRNAs with high coding potential have not been explored. However, we aimed to discover and provide key circRNAs involved in HCC for HCC-related researchers, which still greatly improved our understanding of the underlying mechanism of circRNAs in HCC.

## Data Availability Statement

The original contributions presented in the study are included in the article/**Supplementary Material**. Further inquiries can be directed to the corresponding author.

## Ethics Statement

Participants gave their written informed consent for the materials to appear in publications without limit on the duration of publication.

## Author Contributions

X-YaH conceived and designed the experiments. Z-LH and X-YaH acquired data, related materials, and analysis tools. Z-LH, X-YaH, JH, analyzed the data. Z-LH, X-YaH, X-YuH, Y-HX, and JZ wrote the paper. Y-HX, JZ, and Z-YT revised the paper. All authors contributed to the article and approved the submitted version.

## Funding

This study was supported by grants from the medical-engineering cross fund of Shanghai Jiao Tong University (No. YG2017MS13), the International Foundation of Translational Medicine for abroad Scholars and Students, U.S. and China (No. UCTMP2015-03C001), the National Natural Science Foundation of China (No. 81272401), and the pre-research fund of Shanghai sixth People’s Hospital (LYZY-0229).

## Conflict of Interest

The authors declare that the research was conducted in the absence of any commercial or financial relationships that could be construed as a potential conflict of interest.

## Abbreviations

circRNA, circular RNAs; HCC, hepatocellular carcinoma; ncRNA, non-coding RNAs; PVTT, portal vein tumor thrombosis; GSEA, gene set enrichment analysis; PCC, Pearson correlation coefficients.
